# Tetra­kis(acetonitrile-κ*N*)lithium hexa­fluoridophosphate acetonitrile monosolvate

**DOI:** 10.1107/S1600536811027528

**Published:** 2011-07-30

**Authors:** Daniel M. Seo, Paul D. Boyle, Wesley A. Henderson

**Affiliations:** a Department of Chemical & Biomolecular Engineering, North Carolina State University, Raleigh, NC 27695, USA; b Department of Chemistry, North Carolina State University, Raleigh, NC 27695, USA

## Abstract

In the title compound, [Li(CH_3_CN)_4_]PF_6_·CH_3_CN, the asymmetric unit consists of three independent tetra­hedral [Li(CH_3_CN)_4_]^+^ cations, three uncoordinated PF_6_^−^ anions and three uncoordinated CH_3_CN solvent mol­ecules. The three anions are disordered over two sites through a rotation along one of the F—P—F axes. The relative occupancies of the two sites for the F atoms are 0.643 (16):0.357 (16), 0.677 (10):0.323 (10) and 0.723 (13):0.277 (13). The crystal used was a racemic twin, with approximately equal twin components.

## Related literature

For solvates structures with the PF_6_^−^ anion, see: Zavalij *et al.* (2004 ▶); Armstrong *et al.* (1998 ▶); Black *et al.* (1995 ▶). For solvate structures of CH_3_CN with lithium salts, see: Seo *et al.* (2011*a* ▶,*b* ▶); Klapötke *et al.* (2006 ▶); Brooks *et al.* (2002 ▶); Yokota *et al.* (1999 ▶); Raston *et al.* (1989 ▶).
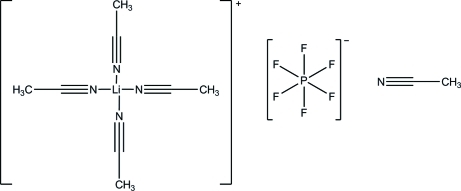


## Experimental

### 

#### Crystal data

[Li(C_2_H_3_N)_4_]PF_6_·C_2_H_3_N
*M* *_r_* = 357.18
Orthorhombic, 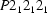

*a* = 8.6064 (3) Å
*b* = 21.9864 (8) Å
*c* = 27.8721 (10) Å
*V* = 5274.1 (3) Å^3^
*Z* = 12
Mo *K*α radiation
μ = 0.22 mm^−1^
*T* = 110 K
0.67 × 0.40 × 0.27 mm

#### Data collection

Bruker–Nonius Kappa Axis X8 APEXII diffractometer
Absorption correction: multi-scan (*SADABS*; Bruker, 2009 ▶) *T*_min_ = 0.870, *T*_max_ = 0.944
128974 measured reflections
17572 independent reflections
13189 reflections with *I* > 2σ(*I*)
*R*_int_ = 0.060

#### Refinement

*R*[*F* ^2^ > 2σ(*F* ^2^)] = 0.054
*wR*(*F* ^2^) = 0.157
*S* = 1.03
17572 reflections
749 parameters
H-atom parameters constrained
Δρ_max_ = 0.46 e Å^−3^
Δρ_min_ = −0.31 e Å^−3^
Absolute structure: Flack (1983 ▶), 7916 Friedel pairs
Flack parameter: 0.45 (7)

### 

Data collection: *APEX2* (Bruker, 2009 ▶); cell refinement: *SAINT* (Bruker, 2009 ▶); data reduction: *SAINT*; program(s) used to solve structure: *SHELXS97* (Sheldrick, 2008 ▶); program(s) used to refine structure: *SHELXTL* (Sheldrick, 2008 ▶); molecular graphics: *ORTEP-3* (Farrugia, 1997 ▶); software used to prepare material for publication: cif2tables.py (Boyle, 2008 ▶).

## Supplementary Material

Crystal structure: contains datablock(s) I, global. DOI: 10.1107/S1600536811027528/fj2439sup1.cif

Structure factors: contains datablock(s) I. DOI: 10.1107/S1600536811027528/fj2439Isup2.hkl

Additional supplementary materials:  crystallographic information; 3D view; checkCIF report

## Figures and Tables

**Fig. 1.**
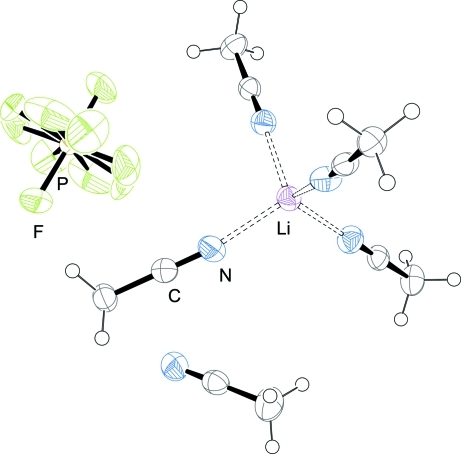
Molecular structure of the title compound. The thermal ellipsoids are shown at a 50% probability level.

**Fig. 2.**
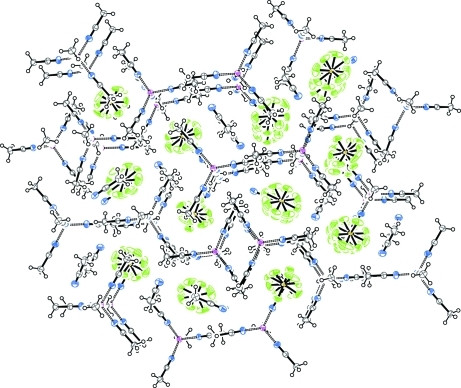
Packing diagram for the title compound (Li-purple; P-gold; F-green; N-blue).

## supplementary crystallographic information

### Comment

LiPF_6_ is the most widely used lithium salt for electrolytes in commercial Li-ion batteries. Little information is available regarding the interactions of the ions and solvent molecules within electrolytes. Crystal structures, however, provide useful models for these interactions. The structure of [Li(CH_3_CN)_4_]PF_6._CH_3_CN is therefore reported here as part of an extensive study exploring solvate structures present in nitrile mixtures with lithium salts.

### Experimental

LiPF_6_ (99.99%) was purchased from Sigma-Aldrich and used as-received. Anhydrous acetonitrile (Sigma Aldrich, 99.8%) was used as-received. In a Vacuum Atmospheres inert atmosphere (N_2_) glove box (< 5 p.p.m. H_2_O), LiPF_6_ (0.2 mmol) and acetonitrile (2 mmol) were sealed in a vial and heated on a hot plate to form a homogeneous solution. Upon standing at ambient temperature, colorless plate single crystals suitable for analysis formed.

### Refinement

The structure was solved by direct methods using the XS program. All non-hydrogen atoms were obtained from the initial solution. The hydrogen atoms were introduced at idealized positions and were allowed to ride on the parent atom. The PF_6_^-^ counterions exhibited an orientational disorder over two sites among the four equatorial fluorine atoms. The occupancies for the primary orientation were 0.643 (16), 0.677 (10), and 0.723 (13) for the three anionic sites, respectively. The structural model was fit to the data using full matrix least-squares based on *F*^2^. The calculated structure factors included corrections for anomalous dispersion from the usual tabulation. The structure was refined using the XL program from *SHELXTL* and graphic plots were produced using the *ORTEP-3* crystallographic program suite.

### Crystal data

**Table:**

[Li(C_2_H_3_N)_4_]PF_6_·C_2_H_3_N	*F*(000) = 2184
*M**_r_* = 357.18	*D*_x_ = 1.349 Mg m^−^^3^
Orthorhombic, *P*2_1_2_1_2_1_	Mo *K*α radiation, λ = 0.71073 Å
Hall symbol: P 2ac 2ab	Cell parameters from 9760 reflections
*a* = 8.6064 (3) Å	θ = 2.9–25.3°
*b* = 21.9864 (8) Å	µ = 0.22 mm^−^^1^
*c* = 27.8721 (10) Å	*T* = 110 K
*V* = 5274.1 (3) Å^3^	Prism, colourless
*Z* = 12	0.67 × 0.40 × 0.27 mm

### Data collection

**Table:**

Bruker–Nonius Kappa Axis X8 APEXII diffractometer	17572 independent reflections
Radiation source: fine-focus sealed tube	13189 reflections with *I* > 2σ(*I*)
graphite	*R*_int_ = 0.060
ω and φ scans	θ_max_ = 31.6°, θ_min_ = 2.0°
Absorption correction: multi-scan (*SADABS*; Bruker, 2009)	*h* = −12→12
*T*_min_ = 0.870, *T*_max_ = 0.944	*k* = −32→32
128974 measured reflections	*l* = −40→40

### Refinement

**Table:**

Refinement on *F*^2^	Secondary atom site location: difference Fourier map
Least-squares matrix: full	Hydrogen site location: inferred from neighbouring sites
*R*[*F*^2^ > 2σ(*F*^2^)] = 0.054	H-atom parameters constrained
*wR*(*F*^2^) = 0.157	*w* = 1/[σ^2^(*F*_o_^2^) + (0.0925*P*)^2^ + 0.5839*P*] where *P* = (*F*_o_^2^ + 2*F*_c_^2^)/3
*S* = 1.03	(Δ/σ)_max_ = 0.001
17572 reflections	Δρ_max_ = 0.46 e Å^−^^3^
749 parameters	Δρ_min_ = −0.31 e Å^−^^3^
0 restraints	Absolute structure: Flack (1983), 7916 Friedel pairs
Primary atom site location: structure-invariant direct methods	Flack parameter: 0.45 (7)

### Special details

**Table:**

Geometry. All e.s.d.'s (except the e.s.d. in the dihedral angle between two l.s. planes) are estimated using the full covariance matrix. The cell e.s.d.'s are taken into account individually in the estimation of e.s.d.'s in distances, angles and torsion angles; correlations between e.s.d.'s in cell parameters are only used when they are defined by crystal symmetry. An approximate (isotropic) treatment of cell e.s.d.'s is used for estimating e.s.d.'s involving l.s. planes.
Refinement. Refinement of *F*^2^ against ALL reflections. The weighted *R*-factor *wR* and goodness of fit *S* are based on *F*^2^, conventional *R*-factors *R* are based on *F*, with *F* set to zero for negative *F*^2^. The threshold expression of *F*^2^ > σ(*F*^2^) is used only for calculating *R*-factors(gt) *etc*. and is not relevant to the choice of reflections for refinement. *R*-factors based on *F*^2^ are statistically about twice as large as those based on *F*, and *R*- factors based on ALL data will be even larger.

### Fractional atomic coordinates and isotropic or equivalent isotropic displacement parameters (Å^2^)

**Table:**

	*x*	*y*	*z*	*U*_iso_*/*U*_eq_	Occ. (<1)
Li1	0.4767 (5)	0.24443 (16)	0.92740 (13)	0.0283 (7)	
N1	0.3918 (2)	0.24237 (9)	0.86018 (7)	0.0315 (4)	
C1	0.3294 (3)	0.24034 (10)	0.82412 (8)	0.0270 (4)	
C2	0.2510 (3)	0.23804 (13)	0.77846 (9)	0.0395 (6)	
H2A	0.2308	0.2795	0.7672	0.059*	
H2B	0.1524	0.2163	0.7820	0.059*	
H2C	0.3166	0.2168	0.7551	0.059*	
N2	0.3962 (3)	0.32101 (8)	0.96007 (7)	0.0309 (4)	
C3	0.3370 (3)	0.35994 (9)	0.97954 (8)	0.0268 (4)	
C4	0.2623 (3)	0.40922 (12)	1.00584 (9)	0.0373 (5)	
H4A	0.1497	0.4026	1.0064	0.056*	
H4B	0.2850	0.4480	0.9900	0.056*	
H4C	0.3019	0.4102	1.0388	0.056*	
N3	0.7092 (3)	0.23919 (9)	0.93095 (7)	0.0337 (4)	
C5	0.8413 (3)	0.24082 (10)	0.93095 (8)	0.0273 (4)	
C6	1.0104 (3)	0.24217 (11)	0.93078 (9)	0.0333 (5)	
H6A	1.0480	0.2546	0.9625	0.050*	
H6B	1.0466	0.2712	0.9066	0.050*	
H6C	1.0504	0.2016	0.9232	0.050*	
N4	0.3809 (2)	0.17257 (9)	0.96466 (7)	0.0317 (4)	
C7	0.3163 (3)	0.13237 (9)	0.98013 (8)	0.0272 (4)	
C8	0.2329 (3)	0.08021 (12)	0.99953 (9)	0.0355 (5)	
H8A	0.1609	0.0645	0.9753	0.053*	
H8B	0.1745	0.0927	1.0281	0.053*	
H8C	0.3073	0.0483	1.0083	0.053*	
Li2	0.4831 (5)	0.07850 (16)	0.55254 (14)	0.0317 (8)	
N5	0.5941 (2)	0.08137 (10)	0.48759 (7)	0.0362 (4)	
C9	0.6478 (3)	0.08505 (10)	0.45077 (8)	0.0294 (4)	
C10	0.7130 (3)	0.08955 (12)	0.40250 (8)	0.0344 (5)	
H10A	0.6803	0.0543	0.3835	0.052*	
H10B	0.8266	0.0905	0.4045	0.052*	
H10C	0.6757	0.1269	0.3871	0.052*	
N6	0.2498 (3)	0.07967 (9)	0.54441 (7)	0.0335 (4)	
C11	0.1191 (3)	0.07718 (10)	0.54844 (8)	0.0289 (4)	
C12	−0.0483 (3)	0.07269 (12)	0.55341 (10)	0.0392 (6)	
H12A	−0.0962	0.0714	0.5215	0.059*	
H12B	−0.0873	0.1081	0.5710	0.059*	
H12C	−0.0745	0.0355	0.5711	0.059*	
N7	0.5280 (3)	0.15207 (10)	0.59300 (8)	0.0391 (5)	
C13	0.5289 (3)	0.19443 (10)	0.61689 (8)	0.0316 (5)	
C14	0.5297 (3)	0.24825 (11)	0.64738 (9)	0.0378 (5)	
H14A	0.4770	0.2391	0.6777	0.057*	
H14B	0.4755	0.2815	0.6311	0.057*	
H14C	0.6373	0.2604	0.6539	0.057*	
N8	0.5384 (3)	0.00158 (9)	0.58959 (8)	0.0386 (5)	
C15	0.5484 (3)	−0.04007 (10)	0.61326 (8)	0.0311 (5)	
C16	0.5610 (4)	−0.09365 (11)	0.64400 (9)	0.0395 (6)	
H16A	0.6246	−0.0838	0.6721	0.059*	
H16B	0.6098	−0.1269	0.6260	0.059*	
H16C	0.4571	−0.1062	0.6545	0.059*	
Li3	0.0076 (4)	0.09502 (17)	0.27226 (13)	0.0285 (7)	
N9	0.0996 (2)	0.17062 (9)	0.24202 (7)	0.0297 (4)	
C17	0.1786 (3)	0.20528 (9)	0.22427 (8)	0.0269 (4)	
C18	0.2810 (3)	0.24923 (11)	0.20127 (9)	0.0366 (5)	
H18A	0.3334	0.2736	0.2259	0.055*	
H18B	0.2197	0.2760	0.1805	0.055*	
H18C	0.3587	0.2277	0.1820	0.055*	
N10	0.0918 (3)	0.01963 (9)	0.23909 (7)	0.0337 (4)	
C19	0.1617 (3)	−0.01773 (10)	0.22136 (9)	0.0306 (5)	
C20	0.2520 (4)	−0.06494 (12)	0.19745 (12)	0.0451 (7)	
H20A	0.1834	−0.0893	0.1770	0.068*	
H20B	0.3006	−0.0912	0.2216	0.068*	
H20C	0.3328	−0.0460	0.1777	0.068*	
N11	0.0944 (2)	0.09247 (9)	0.33925 (6)	0.0298 (4)	
C21	0.1661 (2)	0.09051 (9)	0.37360 (7)	0.0255 (4)	
C22	0.2588 (3)	0.08770 (12)	0.41673 (8)	0.0368 (5)	
H22A	0.3248	0.0514	0.4158	0.055*	
H22B	0.1903	0.0857	0.4448	0.055*	
H22C	0.3242	0.1241	0.4189	0.055*	
N12	−0.2267 (2)	0.09405 (10)	0.27100 (7)	0.0338 (4)	
C23	−0.3580 (3)	0.09458 (10)	0.27519 (7)	0.0280 (4)	
C24	−0.5249 (3)	0.09527 (12)	0.28170 (9)	0.0350 (5)	
H24A	−0.5552	0.0623	0.3034	0.053*	
H24B	−0.5761	0.0896	0.2506	0.053*	
H24C	−0.5565	0.1344	0.2955	0.053*	
P1	0.96424 (7)	0.41157 (2)	0.868437 (19)	0.02657 (11)	
F1	1.0595 (2)	0.35676 (7)	0.84498 (6)	0.0487 (4)	
F2	0.8678 (2)	0.46607 (7)	0.89150 (6)	0.0458 (4)	
F3	0.8398 (7)	0.4121 (3)	0.82520 (16)	0.0585 (15)	0.643 (16)
F4	0.8495 (5)	0.3634 (2)	0.8934 (3)	0.0600 (16)	0.643 (16)
F5	1.0745 (8)	0.4087 (3)	0.91135 (16)	0.0769 (19)	0.643 (16)
F6	1.0657 (7)	0.4576 (3)	0.8407 (3)	0.068 (2)	0.643 (16)
F3'	0.8304 (12)	0.3874 (7)	0.8409 (8)	0.096 (6)	0.357 (16)
F4'	0.924 (3)	0.3725 (5)	0.9132 (5)	0.098 (7)	0.357 (16)
F5'	1.1177 (11)	0.4365 (8)	0.8979 (6)	0.099 (6)	0.357 (16)
F6'	1.027 (3)	0.4544 (7)	0.8274 (6)	0.110 (7)	0.357 (16)
P2	0.51359 (7)	0.42066 (2)	0.15009 (2)	0.02640 (12)	
F7	0.3841 (2)	0.46339 (8)	0.17316 (7)	0.0521 (4)	
F8	0.6435 (2)	0.37953 (9)	0.12640 (9)	0.0696 (6)	
F9	0.3792 (3)	0.37675 (13)	0.12999 (14)	0.0440 (11)	0.677 (10)
F10	0.5018 (5)	0.45856 (15)	0.10114 (11)	0.0616 (13)	0.677 (10)
F11	0.6433 (3)	0.46452 (16)	0.16889 (15)	0.0532 (15)	0.677 (10)
F12	0.5178 (6)	0.38244 (17)	0.19771 (12)	0.0787 (17)	0.677 (10)
F9'	0.4171 (14)	0.4138 (7)	0.1035 (3)	0.117 (7)	0.323 (10)
F10'	0.6068 (14)	0.4797 (3)	0.1342 (6)	0.114 (7)	0.323 (10)
F11'	0.6121 (11)	0.4248 (5)	0.1995 (3)	0.095 (5)	0.323 (10)
F12'	0.4302 (12)	0.3621 (3)	0.1656 (5)	0.101 (6)	0.323 (10)
P3	0.02195 (7)	0.23730 (3)	0.64355 (2)	0.02811 (12)	
F13	0.1488 (2)	0.28030 (9)	0.66797 (7)	0.0571 (5)	
F14	−0.1073 (3)	0.19621 (9)	0.61921 (9)	0.0726 (6)	
F15	0.1179 (6)	0.18006 (16)	0.6596 (2)	0.0811 (17)	0.723 (13)
F16	0.1159 (5)	0.2400 (3)	0.59470 (14)	0.0794 (15)	0.723 (13)
F17	−0.0706 (4)	0.2365 (2)	0.69211 (11)	0.0747 (16)	0.723 (13)
F18	−0.0725 (4)	0.29610 (12)	0.62768 (14)	0.0517 (13)	0.723 (13)
F15'	0.1477 (11)	0.1915 (6)	0.6288 (7)	0.086 (6)	0.277 (13)
F16'	0.044 (2)	0.2731 (7)	0.5971 (4)	0.103 (9)	0.277 (13)
F17'	0.008 (2)	0.1978 (7)	0.6921 (3)	0.098 (7)	0.277 (13)
F18'	−0.1088 (11)	0.2809 (6)	0.6638 (7)	0.109 (11)	0.277 (13)
N13	0.5640 (3)	0.14595 (11)	0.74120 (10)	0.0485 (6)	
C25	0.6588 (3)	0.11557 (12)	0.72554 (10)	0.0390 (6)	
C26	0.7796 (4)	0.07672 (16)	0.70644 (13)	0.0580 (8)	
H26A	0.8673	0.0756	0.7289	0.087*	
H26B	0.8147	0.0927	0.6755	0.087*	
H26C	0.7387	0.0355	0.7021	0.087*	
N14	0.5132 (3)	0.01637 (10)	0.93295 (9)	0.0445 (5)	
C27	0.5128 (3)	0.04165 (11)	0.89718 (10)	0.0363 (5)	
C28	0.5129 (3)	0.07287 (14)	0.85183 (11)	0.0465 (6)	
H28A	0.4534	0.0492	0.8284	0.070*	
H28B	0.6200	0.0776	0.8405	0.070*	
H28C	0.4651	0.1130	0.8557	0.070*	
N15	0.9699 (3)	0.17657 (10)	0.46114 (9)	0.0464 (5)	
C29	0.9032 (3)	0.21382 (12)	0.47998 (9)	0.0359 (5)	
C30	0.8206 (5)	0.26237 (18)	0.50378 (12)	0.0642 (10)	
H30A	0.8950	0.2891	0.5200	0.096*	
H30B	0.7489	0.2451	0.5275	0.096*	
H30C	0.7617	0.2858	0.4800	0.096*	

### Atomic displacement parameters (Å^2^)

**Table:**

	*U*^11^	*U*^22^	*U*^33^	*U*^12^	*U*^13^	*U*^23^
Li1	0.0291 (19)	0.0287 (16)	0.0272 (16)	0.0051 (16)	−0.0016 (15)	−0.0035 (13)
N1	0.0311 (10)	0.0324 (9)	0.0310 (9)	−0.0023 (8)	−0.0011 (8)	0.0010 (7)
C1	0.0247 (10)	0.0278 (9)	0.0284 (10)	−0.0022 (8)	0.0026 (8)	0.0027 (8)
C2	0.0406 (14)	0.0480 (14)	0.0299 (11)	−0.0012 (12)	−0.0090 (10)	−0.0003 (10)
N2	0.0372 (11)	0.0283 (9)	0.0271 (9)	0.0009 (8)	0.0027 (8)	0.0028 (7)
C3	0.0287 (11)	0.0272 (9)	0.0245 (9)	−0.0010 (8)	0.0018 (8)	0.0033 (8)
C4	0.0400 (14)	0.0356 (12)	0.0363 (11)	0.0071 (11)	0.0062 (10)	−0.0050 (10)
N3	0.0324 (11)	0.0371 (10)	0.0317 (9)	−0.0005 (9)	−0.0009 (8)	0.0028 (8)
C5	0.0310 (12)	0.0257 (9)	0.0251 (9)	0.0028 (9)	0.0020 (8)	0.0041 (8)
C6	0.0283 (12)	0.0332 (10)	0.0385 (11)	0.0040 (10)	0.0001 (9)	0.0034 (9)
N4	0.0355 (11)	0.0284 (9)	0.0311 (9)	0.0000 (8)	−0.0012 (8)	0.0016 (7)
C7	0.0278 (11)	0.0283 (10)	0.0254 (9)	0.0073 (8)	−0.0005 (8)	−0.0009 (8)
C8	0.0355 (13)	0.0343 (12)	0.0368 (12)	−0.0045 (10)	0.0047 (10)	0.0055 (9)
Li2	0.036 (2)	0.0274 (16)	0.0315 (17)	−0.0011 (17)	−0.0026 (16)	−0.0001 (13)
N5	0.0297 (10)	0.0480 (12)	0.0309 (9)	−0.0011 (9)	−0.0018 (8)	0.0020 (9)
C9	0.0235 (10)	0.0337 (11)	0.0310 (10)	0.0021 (9)	−0.0035 (8)	0.0005 (9)
C10	0.0336 (12)	0.0372 (11)	0.0324 (11)	0.0017 (10)	0.0033 (9)	0.0008 (10)
N6	0.0360 (11)	0.0303 (9)	0.0343 (10)	−0.0020 (8)	−0.0017 (8)	0.0015 (8)
C11	0.0349 (12)	0.0240 (9)	0.0280 (10)	−0.0012 (9)	−0.0019 (9)	0.0017 (8)
C12	0.0292 (12)	0.0379 (12)	0.0506 (14)	0.0010 (10)	−0.0011 (11)	0.0087 (10)
N7	0.0407 (12)	0.0360 (10)	0.0405 (11)	−0.0043 (10)	−0.0059 (10)	−0.0038 (8)
C13	0.0311 (12)	0.0330 (10)	0.0306 (10)	−0.0027 (10)	−0.0009 (9)	0.0043 (8)
C14	0.0460 (14)	0.0312 (10)	0.0361 (11)	0.0006 (11)	−0.0020 (11)	−0.0048 (9)
N8	0.0426 (12)	0.0353 (10)	0.0378 (10)	0.0028 (10)	−0.0007 (10)	0.0029 (8)
C15	0.0357 (12)	0.0316 (10)	0.0261 (9)	0.0036 (9)	0.0025 (9)	−0.0033 (8)
C16	0.0546 (16)	0.0326 (11)	0.0313 (11)	0.0068 (11)	0.0075 (11)	0.0061 (9)
Li3	0.0279 (19)	0.0322 (16)	0.0255 (15)	−0.0030 (16)	0.0004 (14)	0.0003 (14)
N9	0.0325 (10)	0.0310 (9)	0.0257 (9)	0.0017 (8)	−0.0010 (8)	0.0007 (7)
C17	0.0307 (11)	0.0265 (9)	0.0235 (9)	0.0074 (9)	−0.0027 (8)	−0.0025 (8)
C18	0.0416 (14)	0.0318 (11)	0.0366 (12)	−0.0035 (10)	0.0091 (11)	0.0010 (9)
N10	0.0369 (11)	0.0293 (9)	0.0348 (10)	0.0018 (8)	−0.0004 (9)	0.0012 (7)
C19	0.0292 (11)	0.0290 (10)	0.0336 (11)	−0.0014 (9)	0.0018 (10)	0.0042 (8)
C20	0.0441 (16)	0.0304 (12)	0.0607 (17)	0.0055 (11)	0.0197 (13)	0.0022 (11)
N11	0.0297 (9)	0.0334 (9)	0.0264 (8)	0.0001 (8)	−0.0012 (7)	0.0023 (7)
C21	0.0243 (10)	0.0250 (8)	0.0272 (9)	−0.0001 (8)	0.0044 (8)	0.0000 (8)
C22	0.0350 (12)	0.0425 (13)	0.0329 (11)	−0.0007 (11)	−0.0081 (10)	−0.0022 (10)
N12	0.0301 (10)	0.0391 (10)	0.0322 (9)	0.0002 (9)	0.0004 (8)	0.0001 (8)
C23	0.0314 (12)	0.0284 (9)	0.0241 (9)	−0.0002 (9)	−0.0011 (8)	−0.0008 (8)
C24	0.0276 (11)	0.0402 (11)	0.0373 (11)	−0.0002 (10)	0.0007 (10)	−0.0009 (9)
P1	0.0314 (3)	0.0229 (2)	0.0254 (2)	0.0009 (2)	−0.0007 (2)	−0.00043 (19)
F1	0.0530 (10)	0.0338 (7)	0.0593 (10)	0.0085 (7)	0.0162 (8)	−0.0081 (7)
F2	0.0536 (10)	0.0312 (7)	0.0526 (9)	0.0058 (7)	0.0095 (8)	−0.0099 (7)
F3	0.054 (2)	0.081 (4)	0.0402 (18)	0.017 (3)	−0.0215 (14)	−0.0149 (19)
F4	0.054 (2)	0.0334 (15)	0.092 (4)	−0.0029 (15)	0.031 (2)	0.012 (2)
F5	0.083 (4)	0.099 (4)	0.048 (2)	0.014 (3)	−0.044 (2)	−0.006 (2)
F6	0.052 (2)	0.0342 (19)	0.118 (6)	−0.0092 (16)	0.040 (3)	0.018 (3)
F3'	0.037 (3)	0.083 (8)	0.169 (16)	0.000 (5)	−0.033 (7)	−0.080 (9)
F4'	0.174 (17)	0.050 (5)	0.070 (6)	0.045 (7)	0.087 (8)	0.036 (4)
F5'	0.040 (4)	0.120 (9)	0.137 (10)	0.033 (5)	−0.038 (5)	−0.103 (8)
F6'	0.210 (18)	0.050 (6)	0.070 (7)	0.041 (9)	0.089 (9)	0.028 (4)
P2	0.0241 (3)	0.0235 (2)	0.0317 (3)	0.0000 (2)	−0.0001 (2)	−0.00195 (19)
F7	0.0391 (9)	0.0438 (9)	0.0732 (12)	0.0021 (7)	0.0123 (9)	−0.0213 (8)
F8	0.0347 (9)	0.0619 (11)	0.1122 (17)	0.0078 (9)	0.0047 (10)	−0.0483 (12)
F9	0.0313 (12)	0.0368 (15)	0.064 (2)	−0.0071 (10)	0.0013 (13)	−0.0184 (14)
F10	0.079 (3)	0.061 (2)	0.0447 (15)	−0.0105 (19)	0.0114 (16)	0.0156 (13)
F11	0.0272 (12)	0.048 (2)	0.085 (3)	−0.0038 (12)	−0.0091 (15)	−0.033 (2)
F12	0.120 (4)	0.064 (2)	0.0523 (17)	0.007 (3)	−0.024 (2)	0.0250 (16)
F9'	0.109 (9)	0.177 (16)	0.064 (5)	0.048 (12)	−0.043 (6)	−0.033 (8)
F10'	0.098 (9)	0.044 (4)	0.200 (17)	−0.015 (4)	0.075 (11)	0.031 (6)
F11'	0.088 (6)	0.137 (11)	0.060 (5)	0.028 (7)	−0.035 (5)	−0.023 (6)
F12'	0.097 (8)	0.045 (4)	0.160 (15)	−0.029 (4)	0.055 (10)	0.004 (5)
P3	0.0297 (3)	0.0256 (2)	0.0290 (2)	0.0024 (2)	−0.0014 (2)	0.00179 (19)
F13	0.0469 (10)	0.0572 (10)	0.0673 (12)	−0.0074 (9)	−0.0192 (9)	−0.0042 (9)
F14	0.0601 (13)	0.0539 (11)	0.1037 (17)	−0.0136 (10)	−0.0200 (12)	−0.0275 (11)
F15	0.087 (3)	0.0463 (16)	0.110 (4)	0.0323 (18)	−0.012 (3)	0.024 (2)
F16	0.080 (2)	0.112 (4)	0.0463 (17)	0.023 (2)	0.0308 (16)	0.004 (2)
F17	0.073 (2)	0.106 (4)	0.0446 (15)	0.003 (3)	0.0289 (15)	0.0200 (19)
F18	0.059 (2)	0.0370 (12)	0.059 (2)	0.0187 (12)	−0.0195 (18)	0.0046 (12)
F15'	0.044 (4)	0.073 (8)	0.140 (14)	0.017 (5)	0.009 (6)	−0.049 (9)
F16'	0.151 (18)	0.098 (10)	0.061 (6)	−0.075 (12)	−0.047 (10)	0.040 (7)
F17'	0.136 (15)	0.100 (10)	0.059 (5)	−0.060 (11)	−0.015 (7)	0.024 (6)
F18'	0.048 (5)	0.099 (12)	0.18 (2)	0.020 (5)	−0.014 (7)	−0.105 (15)
N13	0.0464 (14)	0.0421 (12)	0.0570 (15)	−0.0026 (11)	−0.0012 (12)	−0.0039 (11)
C25	0.0411 (14)	0.0352 (11)	0.0406 (13)	−0.0076 (11)	−0.0069 (11)	0.0036 (10)
C26	0.0548 (19)	0.0545 (17)	0.065 (2)	0.0112 (16)	0.0057 (16)	−0.0071 (15)
N14	0.0473 (14)	0.0399 (11)	0.0462 (12)	−0.0034 (10)	−0.0048 (11)	−0.0132 (9)
C27	0.0263 (11)	0.0322 (10)	0.0504 (14)	−0.0019 (9)	−0.0028 (11)	−0.0155 (10)
C28	0.0307 (12)	0.0523 (15)	0.0566 (16)	−0.0006 (12)	−0.0023 (12)	0.0015 (12)
N15	0.0504 (14)	0.0382 (11)	0.0507 (13)	0.0017 (11)	−0.0040 (12)	−0.0021 (10)
C29	0.0339 (13)	0.0417 (13)	0.0321 (11)	0.0010 (10)	−0.0016 (10)	0.0077 (10)
C30	0.074 (2)	0.076 (2)	0.0431 (16)	0.036 (2)	0.0067 (16)	0.0003 (16)

### Geometric parameters (Å, °)

**Table:**

Li1—N3	2.007 (5)	C20—H20A	0.9800
Li1—N1	2.012 (4)	C20—H20B	0.9800
Li1—N2	2.036 (4)	C20—H20C	0.9800
Li1—N4	2.063 (4)	N11—C21	1.140 (3)
N1—C1	1.141 (3)	C21—C22	1.444 (3)
C1—C2	1.441 (3)	C22—H22A	0.9800
C2—H2A	0.9800	C22—H22B	0.9800
C2—H2B	0.9800	C22—H22C	0.9800
C2—H2C	0.9800	N12—C23	1.136 (3)
N2—C3	1.134 (3)	C23—C24	1.448 (3)
C3—C4	1.458 (3)	C24—H24A	0.9800
C4—H4A	0.9800	C24—H24B	0.9800
C4—H4B	0.9800	C24—H24C	0.9800
C4—H4C	0.9800	P1—F3'	1.484 (10)
N3—C5	1.137 (3)	P1—F5	1.528 (5)
C5—C6	1.456 (3)	P1—F6	1.544 (6)
C6—H6A	0.9800	P1—F4'	1.555 (7)
C6—H6B	0.9800	P1—F6'	1.577 (14)
C6—H6C	0.9800	P1—F2	1.5929 (16)
N4—C7	1.129 (3)	P1—F1	1.5975 (16)
C7—C8	1.457 (3)	P1—F4	1.607 (4)
C8—H8A	0.9800	P1—F3	1.612 (5)
C8—H8B	0.9800	P1—F5'	1.650 (8)
C8—H8C	0.9800	P2—F12'	1.535 (6)
Li2—N7	2.009 (4)	P2—F9'	1.548 (7)
Li2—N6	2.021 (5)	P2—F11	1.565 (3)
Li2—N8	2.038 (4)	P2—F12	1.571 (3)
Li2—N5	2.048 (5)	P2—F8	1.5823 (18)
N5—C9	1.128 (3)	P2—F10'	1.589 (7)
C9—C10	1.461 (3)	P2—F7	1.5933 (17)
C10—H10A	0.9800	P2—F10	1.602 (3)
C10—H10B	0.9800	P2—F9	1.608 (2)
C10—H10C	0.9800	P2—F11'	1.620 (7)
N6—C11	1.132 (3)	P3—F16'	1.526 (8)
C11—C12	1.451 (4)	P3—F15'	1.535 (8)
C12—H12A	0.9800	P3—F15	1.570 (3)
C12—H12B	0.9800	P3—F17	1.571 (3)
C12—H12C	0.9800	P3—F18'	1.582 (8)
N7—C13	1.145 (3)	P3—F16	1.585 (3)
C13—C14	1.457 (3)	P3—F14	1.586 (2)
C14—H14A	0.9800	P3—F18	1.590 (2)
C14—H14B	0.9800	P3—F13	1.5967 (19)
C14—H14C	0.9800	P3—F17'	1.614 (9)
N8—C15	1.132 (3)	N13—C25	1.141 (4)
C15—C16	1.461 (3)	C25—C26	1.447 (4)
C16—H16A	0.9800	C26—H26A	0.9800
C16—H16B	0.9800	C26—H26B	0.9800
C16—H16C	0.9800	C26—H26C	0.9800
Li3—N11	2.012 (4)	N14—C27	1.142 (4)
Li3—N12	2.017 (4)	C27—C28	1.438 (4)
Li3—N9	2.025 (4)	C28—H28A	0.9800
Li3—N10	2.032 (4)	C28—H28B	0.9800
N9—C17	1.135 (3)	C28—H28C	0.9800
C17—C18	1.456 (3)	N15—C29	1.129 (3)
C18—H18A	0.9800	C29—C30	1.444 (4)
C18—H18B	0.9800	C30—H30A	0.9800
C18—H18C	0.9800	C30—H30B	0.9800
N10—C19	1.132 (3)	C30—H30C	0.9800
C19—C20	1.458 (3)		
			
N3—Li1—N1	114.0 (2)	F6—P1—F1	90.0 (3)
N3—Li1—N2	111.4 (2)	F4'—P1—F1	91.5 (3)
N1—Li1—N2	108.14 (19)	F6'—P1—F1	88.7 (6)
N3—Li1—N4	109.27 (19)	F2—P1—F1	179.44 (11)
N1—Li1—N4	107.83 (19)	F3'—P1—F4	60.7 (8)
N2—Li1—N4	105.79 (19)	F5—P1—F4	90.9 (3)
C1—N1—Li1	173.2 (2)	F6—P1—F4	175.3 (4)
N1—C1—C2	179.7 (3)	F6'—P1—F4	156.7 (8)
C1—C2—H2A	109.5	F2—P1—F4	90.04 (19)
C1—C2—H2B	109.5	F1—P1—F4	89.75 (19)
H2A—C2—H2B	109.5	F5—P1—F3	176.3 (3)
C1—C2—H2C	109.5	F6—P1—F3	89.8 (4)
H2A—C2—H2C	109.5	F4'—P1—F3	117.2 (9)
H2B—C2—H2C	109.5	F6'—P1—F3	71.4 (8)
C3—N2—Li1	172.3 (2)	F2—P1—F3	87.1 (2)
N2—C3—C4	178.4 (2)	F1—P1—F3	92.3 (2)
C3—C4—H4A	109.5	F4—P1—F3	85.5 (3)
C3—C4—H4B	109.5	F3'—P1—F5'	177.7 (5)
H4A—C4—H4B	109.5	F6—P1—F5'	65.1 (8)
C3—C4—H4C	109.5	F4'—P1—F5'	87.8 (7)
H4A—C4—H4C	109.5	F6'—P1—F5'	83.6 (8)
H4B—C4—H4C	109.5	F2—P1—F5'	88.0 (3)
C5—N3—Li1	174.2 (2)	F1—P1—F5'	92.5 (3)
N3—C5—C6	179.3 (3)	F4—P1—F5'	119.7 (7)
C5—C6—H6A	109.5	F3—P1—F5'	154.4 (7)
C5—C6—H6B	109.5	F12'—P2—F9'	84.5 (8)
H6A—C6—H6B	109.5	F12'—P2—F11	139.1 (6)
C5—C6—H6C	109.5	F9'—P2—F11	136.3 (6)
H6A—C6—H6C	109.5	F12'—P2—F12	47.5 (5)
H6B—C6—H6C	109.5	F9'—P2—F12	131.9 (6)
C7—N4—Li1	171.2 (2)	F11—P2—F12	91.7 (2)
N4—C7—C8	179.3 (2)	F12'—P2—F8	88.2 (3)
C7—C8—H8A	109.5	F9'—P2—F8	88.5 (4)
C7—C8—H8B	109.5	F11—P2—F8	89.29 (13)
H8A—C8—H8B	109.5	F12—P2—F8	91.75 (18)
C7—C8—H8C	109.5	F12'—P2—F10'	177.5 (6)
H8A—C8—H8C	109.5	F9'—P2—F10'	96.6 (9)
H8B—C8—H8C	109.5	F12—P2—F10'	131.4 (7)
N7—Li2—N6	104.1 (2)	F8—P2—F10'	89.6 (3)
N7—Li2—N8	109.8 (2)	F12'—P2—F7	93.1 (3)
N6—Li2—N8	107.4 (2)	F9'—P2—F7	91.2 (4)
N7—Li2—N5	112.4 (2)	F11—P2—F7	90.05 (12)
N6—Li2—N5	111.3 (2)	F12—P2—F7	89.46 (17)
N8—Li2—N5	111.4 (2)	F8—P2—F7	178.64 (13)
C9—N5—Li2	175.7 (2)	F10'—P2—F7	89.1 (3)
N5—C9—C10	178.4 (2)	F12'—P2—F10	130.2 (6)
C9—C10—H10A	109.5	F9'—P2—F10	45.8 (6)
C9—C10—H10B	109.5	F11—P2—F10	90.6 (2)
H10A—C10—H10B	109.5	F12—P2—F10	177.5 (2)
C9—C10—H10C	109.5	F8—P2—F10	89.23 (16)
H10A—C10—H10C	109.5	F10'—P2—F10	50.9 (6)
H10B—C10—H10C	109.5	F7—P2—F10	89.58 (14)
C11—N6—Li2	167.4 (2)	F11—P2—F9	178.8 (2)
N6—C11—C12	178.9 (3)	F12—P2—F9	89.4 (2)
C11—C12—H12A	109.5	F8—P2—F9	91.13 (11)
C11—C12—H12B	109.5	F10'—P2—F9	139.1 (7)
H12A—C12—H12B	109.5	F7—P2—F9	89.50 (12)
C11—C12—H12C	109.5	F10—P2—F9	88.3 (2)
H12A—C12—H12C	109.5	F12'—P2—F11'	93.0 (7)
H12B—C12—H12C	109.5	F9'—P2—F11'	177.4 (8)
C13—N7—Li2	169.3 (3)	F11—P2—F11'	46.2 (4)
N7—C13—C14	179.8 (3)	F12—P2—F11'	45.6 (4)
C13—C14—H14A	109.5	F8—P2—F11'	91.0 (3)
C13—C14—H14B	109.5	F10'—P2—F11'	85.9 (8)
H14A—C14—H14B	109.5	F7—P2—F11'	89.4 (3)
C13—C14—H14C	109.5	F10—P2—F11'	136.7 (5)
H14A—C14—H14C	109.5	F9—P2—F11'	135.0 (5)
H14B—C14—H14C	109.5	F16'—P3—F15'	91.3 (9)
C15—N8—Li2	169.9 (3)	F16'—P3—F15	126.0 (8)
N8—C15—C16	179.7 (3)	F16'—P3—F17	143.1 (8)
C15—C16—H16A	109.5	F15'—P3—F17	125.5 (8)
C15—C16—H16B	109.5	F15—P3—F17	90.7 (3)
H16A—C16—H16B	109.5	F16'—P3—F18'	94.6 (10)
C15—C16—H16C	109.5	F15'—P3—F18'	174.1 (9)
H16A—C16—H16C	109.5	F15—P3—F18'	139.3 (8)
H16B—C16—H16C	109.5	F17—P3—F18'	48.6 (8)
N11—Li3—N12	112.78 (19)	F15'—P3—F16	55.6 (7)
N11—Li3—N9	105.30 (19)	F15—P3—F16	90.3 (3)
N12—Li3—N9	113.1 (2)	F17—P3—F16	178.5 (3)
N11—Li3—N10	105.50 (19)	F18'—P3—F16	130.3 (8)
N12—Li3—N10	109.9 (2)	F16'—P3—F14	91.1 (4)
N9—Li3—N10	109.91 (18)	F15'—P3—F14	90.3 (4)
C17—N9—Li3	165.3 (2)	F15—P3—F14	91.9 (2)
N9—C17—C18	179.4 (3)	F17—P3—F14	90.36 (18)
C17—C18—H18A	109.5	F18'—P3—F14	90.0 (3)
C17—C18—H18B	109.5	F16—P3—F14	90.7 (2)
H18A—C18—H18B	109.5	F16'—P3—F18	53.8 (8)
C17—C18—H18C	109.5	F15'—P3—F18	145.1 (8)
H18A—C18—H18C	109.5	F15—P3—F18	178.9 (2)
H18B—C18—H18C	109.5	F17—P3—F18	89.4 (2)
C19—N10—Li3	168.7 (2)	F16—P3—F18	89.5 (2)
N10—C19—C20	178.6 (3)	F14—P3—F18	89.17 (14)
C19—C20—H20A	109.5	F16'—P3—F13	88.3 (4)
C19—C20—H20B	109.5	F15'—P3—F13	91.2 (4)
H20A—C20—H20B	109.5	F15—P3—F13	89.64 (19)
C19—C20—H20C	109.5	F17—P3—F13	89.21 (17)
H20A—C20—H20C	109.5	F18'—P3—F13	88.6 (3)
H20B—C20—H20C	109.5	F16—P3—F13	89.73 (19)
C21—N11—Li3	169.0 (2)	F14—P3—F13	178.36 (12)
N11—C21—C22	179.2 (2)	F18—P3—F13	89.25 (13)
C21—C22—H22A	109.5	F16'—P3—F17'	176.8 (9)
C21—C22—H22B	109.5	F15'—P3—F17'	85.7 (8)
H22A—C22—H22B	109.5	F15—P3—F17'	50.9 (7)
C21—C22—H22C	109.5	F18'—P3—F17'	88.5 (9)
H22A—C22—H22C	109.5	F16—P3—F17'	141.2 (7)
H22B—C22—H22C	109.5	F14—P3—F17'	90.0 (4)
C23—N12—Li3	173.0 (2)	F18—P3—F17'	129.3 (7)
N12—C23—C24	178.7 (2)	F13—P3—F17'	90.7 (4)
C23—C24—H24A	109.5	N13—C25—C26	179.1 (3)
C23—C24—H24B	109.5	C25—C26—H26A	109.5
H24A—C24—H24B	109.5	C25—C26—H26B	109.5
C23—C24—H24C	109.5	H26A—C26—H26B	109.5
H24A—C24—H24C	109.5	C25—C26—H26C	109.5
H24B—C24—H24C	109.5	H26A—C26—H26C	109.5
F3'—P1—F5	150.8 (8)	H26B—C26—H26C	109.5
F3'—P1—F6	114.5 (9)	N14—C27—C28	179.3 (3)
F5—P1—F6	93.8 (4)	C27—C28—H28A	109.5
F3'—P1—F4'	92.6 (7)	C27—C28—H28B	109.5
F5—P1—F4'	59.1 (8)	H28A—C28—H28B	109.5
F6—P1—F4'	152.9 (9)	C27—C28—H28C	109.5
F3'—P1—F6'	96.0 (9)	H28A—C28—H28C	109.5
F5—P1—F6'	112.3 (8)	H28B—C28—H28C	109.5
F4'—P1—F6'	171.4 (9)	N15—C29—C30	178.8 (3)
F3'—P1—F2	94.3 (4)	C29—C30—H30A	109.5
F5—P1—F2	92.2 (2)	C29—C30—H30B	109.5
F6—P1—F2	90.2 (3)	H30A—C30—H30B	109.5
F4'—P1—F2	88.6 (3)	C29—C30—H30C	109.5
F6'—P1—F2	91.3 (6)	H30A—C30—H30C	109.5
F3'—P1—F1	85.2 (4)	H30B—C30—H30C	109.5
F5—P1—F1	88.3 (2)		
